# Microbial PAMPs-PRR driven neuroimmune signaling in the development of post-infectious persistent pain

**DOI:** 10.1007/s44254-026-00199-6

**Published:** 2026-10-19

**Authors:** Ying Hong, Gang Wang, Mengmeng Zhou, Yan Xu, Hanbing Wang, Mao Ye, Xiao Xiao, Li Zhou, Guochang Hu, Chunling Jiang

**Affiliations:** 1 Department of Anesthesiology, The Research Units of West China (2018RU012), West China Hospital, Chinese Academy of Medical Sciences, Sichuan University, Chengdu, 610041 China; 2 West China School of Medicine, Sichuan University, Chengdu, 610041 China; 3 Departments of Anesthesiology and Pharmacology & Regenerative Medicine, University of Illinois College of Medicine, Chicago, Illinois USA

**Keywords:** Post-infectious persistent pain, Neuroimmune signaling, Pathogen-associated molecular patterns

## Abstract

Post-infectious persistent pain (PIPP) is a common long-term outcome of infection, but the link between transient infection and persistent pain remains unclear. Traditional explanations, such as tissue injury, residual inflammation, and nerve damage, do not fully explain pain persistence after pathogen clearance. This review proposes that signaling between pathogen-associated molecular patterns and pattern recognition receptors (PAMP–PRR) is a key mechanistic bridge from acute infection to persistent pain. Microbial ligands from bacterial, viral, fungal, and parasitic pathogens activate PRRs in immune cells, sensory neurons, glia, and epithelial cells, triggering shared neuroimmune programs that include inflammatory amplification, neuron–immune crosstalk, glial activation, and sensitization of peripheral and central pain pathways. Persistent microbial products, viral latency, repeated antigen exposure, and trained immunity may maintain these circuits and promote a shift from PAMP-driven responses to damage-associated molecular pattern (DAMP)-mediated sterile neuroinflammation. These observations reframe PIPP as a dynamic neuroimmune disorder and support biomarker-informed, stage-specific therapies aimed at restoring neuroimmune homeostasis while preserving host defense.

**Graphical Abstract:**

**Figure:**
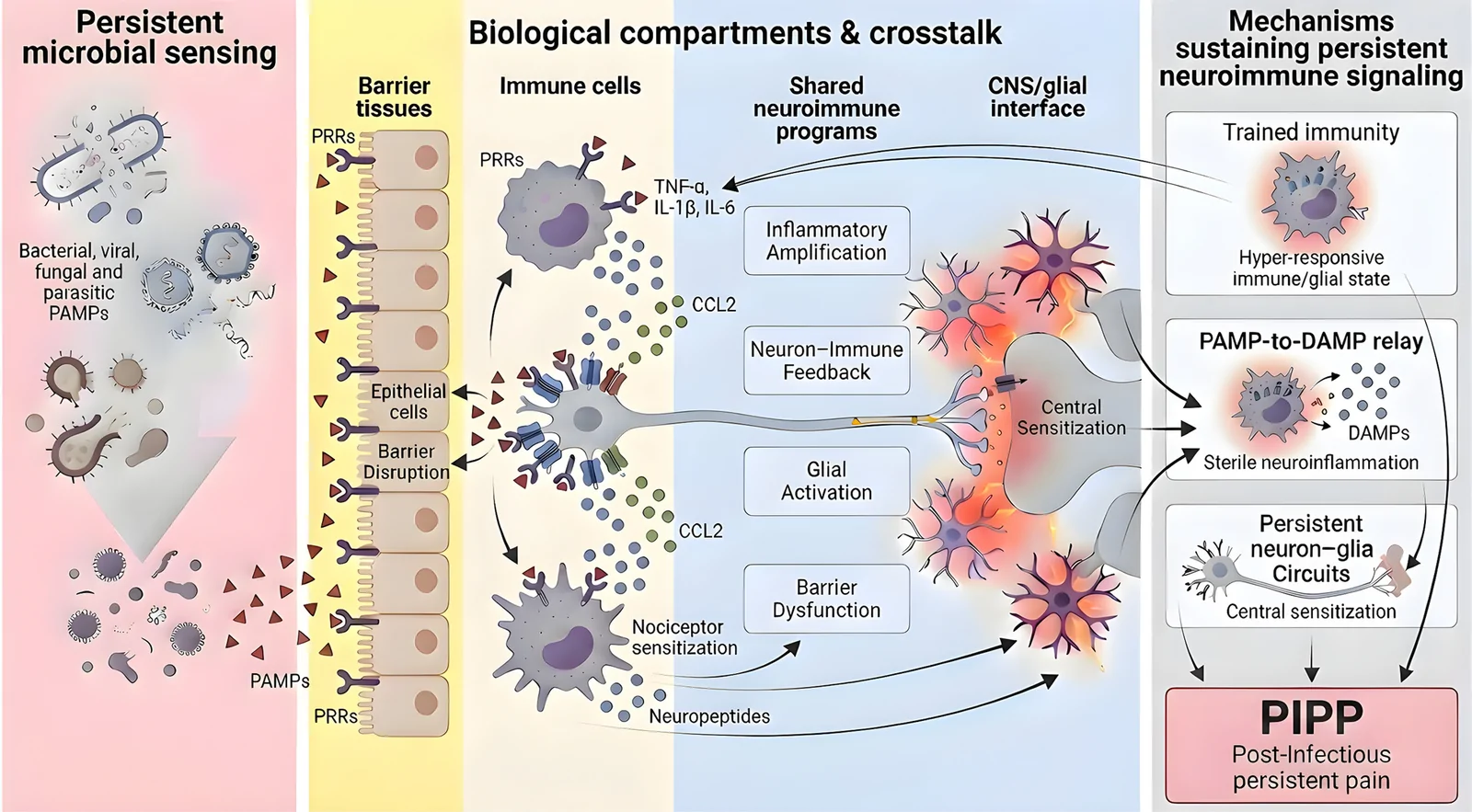

## Introduction

Pain is a common feature of infection and serves a protective role by promoting behaviors that minimize tissue damage and facilitate recovery. Across different post-infectious conditions, however, pain persists beyond the expected resolution of the infection in approximately 10–33% of individuals [1]. Post-infectious persistent pain (PIPP) is used here descriptively to encompass pain that develops during or after infection and remains present or recurrent at least 3 months after the onset of the acute illness, consistent with the IASP/ICD-11 (International Association for the Study of Pain/International Classification of Diseases 11th Revision) criterion for chronic pain and broadly aligned with the temporal criteria applied to various infectious disease, such as postherpetic neuralgia (PHN) and post-COVID-19 condition, although disease-specific definitions vary [2–4]. Regardless of its underlying cause, PIPP imposes substantial functional and psychosocial burdens, reducing quality of life and physical functioning while frequently co-occurring with depression, anxiety, and other affective disorders [5, 6].

During acute infection, the host innate immune system detects invading pathogens through highly conserved molecular structures known as pathogen-associated molecular patterns (PAMPs), including bacterial cell wall components and viral nucleic acids. These microbial signatures are recognized by host pattern recognition receptors (PRRs), which initiate inflammatory signaling cascades essential for pathogen elimination and host defense [7]. Importantly, PRRs are expressed not only by classical immune cells but also by sensory neurons, glial cells, and epithelial cells at barrier surfaces, enabling microbial signals to directly or indirectly reshape nociceptive processing through neuroimmune crosstalk [8–13].

Traditional explanations for PIPP have primarily emphasized residual tissue damage, fibrosis, or irreversible nerve injury. While these mechanisms likely contribute in some cases, they do not adequately explain why pain frequently persists long after pathogen clearance and apparent resolution of inflammation. Increasing evidence instead suggests that persistent neuroimmune signaling initiated by microbial PAMPs and their cognate PRRs provides a key mechanistic bridge linking acute infection to persistent pain [1, 7, 14]. Importantly, implicating PAMP–PRR signaling in PIPP does not imply that persistent pain necessarily reflects ongoing infection. Rather, retained microbial products, long-lasting innate immune reprogramming, and secondary sterile inflammatory pathways may sustain nociceptive sensitization even after the infectious insult has resolved. In this mini-review, we propose that microbial PAMP–PRR signaling constitutes a central mechanistic axis linking transient microbial sensing to persistent neuroimmune activation and ultimately to the development of PIPP. Table 1 summarizes the representative PAMP–PRR interactions and the current evidence supporting their potential contribution to PIPP.

**Table 1:** Evidence summary of PAMP–PRR-mediated neuroimmune signaling in PIPP

**Domain**	**Evidence summary**	**PRRs or signaling nodes**	**Overall confidence**	**Key references**
Immune cells link persistent microbial signaling to nociceptor sensitization	Immune cells sustain pain through inflammasome activation and broader PRR-mediated neuroimmune crosstalk	NLRP3, PRR signaling	High	[7, 12, 14–19]
Sensory neurons detect microbial and immune signals that sustain pain	Sensory neurons detect microbial signals through PRR-linked pathways, promoting persistent peripheral sensitization	TLR4, TLR3, STING, TRPA1	Moderate	[11, 13–15, 20, 21]
Glial cells sustain sterile neuroinflammation and central sensitization	Glial cells can amplify pain after the initial trigger wanes, sustaining sterile neuroinflammation and central sensitization	NLRP3, PRR signaling, DAMPs	Moderate	[10, 12, 19, 22–30]
Epithelial cells prolong peripheral neuroimmune signaling after infection	Barrier-associated cells may prolong local immune activation and nociceptor responsiveness after infection, but evidence remains limited	TLR3	Low	[13]
Persistence of bacterial PAMPs after acute infection	Retained bacterial ligands may persist after acute infection and maintain PRR-dependent inflammatory signaling and nociceptor sensitization	TLR4, TLR5, TLR9, NF-κB	Moderate	[31–36]
Latency and persistent nucleic acid sensing of viral PAMPs after acute infection	Viral latency and persistent nucleic acid sensing may sustain neuroimmune activation after acute infection	STING, IRF3, PRR signaling	Moderate	[20, 30, 37–43]
Post-COVID pain as a viral persistence-associated model	Post-COVID pain is a contemporary model of possible post-infectious pain persistence, supported by symptom studies and persistence hypotheses	TLR4, PRR signaling	Moderate	[3, 5, 6, 44–46]
Fungal ligands as drivers of prolonged neuroimmune imprinting	Fungal ligands may induce durable innate immune activation, but direct links to chronic pain remain limited	PRR signaling, trained immunity	Low	[18, 47–51]
Parasitic ligands as drivers of prolonged neuroimmune imprinting	Parasitic infection may drive durable immune remodeling, but evidence for defined post-infectious pain is sparse	NLRP3, NF-κB, PRR signaling	Low	[52–54]
How PAMP–PRR signaling may outlast infection	Retained ligands, latent pathogen products, trained immunity, and a PAMP-to-DAMP transition may prolong pain-promoting innate signaling after infection	PRR signaling, STING, IRF3, NLRP3, DAMPs, trained immunity	Moderate	[35, 37–41, 44–46, 48, 49, 55]
Therapeutic implications	Therapeutic translation should prioritize mechanism- and timing-guided strategies that promote neuroimmune resolution	PRR signaling, NLRP3, STING, TLR4	Moderate	[3, 12, 20, 21, 56–59]

Certainty of the qualitative evidence: The overall confidence of the qualitative was evidenced as high, moderate, or low. High confidence indicates findings supported by generally robust and consistent evidence with strong biological plausibility. Moderate confidence indicates findings supported by credible but incomplete or partly indirect evidence, or by evidence with some methodological limitations. Low confidence indicates findings for which the evidence is limited, heterogeneous, indirect, or more speculative in relation to PIPP. *DAMP* damage-associated molecular pattern, *IRF3* interferon regulatory factor 3, *NF-κB* nuclear factor kappa B, *NLRP3* NOD-like receptor family pyrin domain-containing protein 3, *PAMP* pathogenassociated molecular pattern, *PIPP* post-infectious persistent pain, *PRR* pattern recognition receptor, *STING* stimulator of interferon genes, *TLR* toll-like receptor, *TRPA1* transient receptor potential ankyrin 1

## PAMP–PRR signaling as a mechanistic bridge from infection to persistent pain

Post-infectious pain does not necessarily indicate active infection. Although tissue damage, fibrosis, and nerve injury may contribute, they do not fully explain pain persistence after pathogen clearance and overt inflammation resolve. Infection-initiated neuroimmune signaling may instead be prolonged by latent pathogens, retained microbial products, trained immunity, or a shift to sterile neuroinflammation in which cytokines and damage-associated molecular patterns (DAMPs) replace microbial ligands as the main PRR activators. This PAMP-to-DAMP relay may create self-sustaining neuroimmune circuits that maintain persistent nociceptive activity. Accordingly, the key question is how different cellular compartments sense, amplify, and translate these signals into persistent nociceptive activity.

### Immune cells translate persistent microbial signals into nociceptor sensitization

Immune cells provide a major interface between infection-related signals and sensory pathways. Through PRRs, they detect persistent pathogens or retained microbial products and initiate inflammatory programs required for host defense. Prior infection can also leave innate immune cells epigenetically and metabolically reprogrammed, leading to exaggerated responses after pathogen clearance. These immune alterations may modulate sensory neurons through cytokine and chemokine release, thereby contributing to nociceptor activation and sensitization [15, 16]. For example, evidence from broader inflammatory and neuropathic pain studies supports roles for tumor necrosis factor (TNF)-α, interleukin (IL)-1β, and IL-6 in nociceptor sensitization and pro-nociceptive signaling, while CCL2 (C-C motif chemokine ligand 2) has been shown to increase the excitability of primary afferents through CCR2/Nav1.8 (C-C chemokine receptor type 2/voltage-gated sodium channel 1.8)-related mechanisms [17, 60]. Thus, the key contribution of immune cells in PIPP is not only pathogen sensing itself, but also sustained immune-to-neural communication that can persist beyond the acute infectious phase. Importantly, immune-mediated sensitization and direct neuronal microbial sensing frequently coexist, rather than representing independent mechanisms.

### Sensory neurons integrate microbial and immune signals

Sensory neurons are a key convergence site for persistent pathogen and persistent microbial debris. In addition to responding to cytokines and chemokines released by immune cells, nociceptors express pattern-recognition receptors and, in some contexts, can directly detect microbial ligands [61, 62], suggesting that microbial signals can, at least in some contexts, activate nociceptors without first being relayed through immune cells. Furthermore, activated nociceptors release neuropeptides and neurotransmitters such as substance P (SP) and calcitonin gene-related peptide (CGRP), which can act on immune cells to modulate inflammatory responses and, in certain settings, immune cell recruitment [63]. These reciprocal interactions form a neuroimmune axis in which immune activation drives nociceptor sensitization, while neural activity in turn shapes immune cell function. Thus, sensory neurons function not only as passive recipients of inflammatory signals but also as active regulators of neuroimmune communication that reinforce persistent nociceptor sensitization and PIPP.

### Glial cells sustain sterile neuroinflammation and central sensitization

Glial cells are key contributors to the transition from microbially triggered signaling to longer-lasting sterile unresolved neuroinflammation, particularly as direct microbial input wanes over time. Their major importance in PIPP lies in sustaining pathological neuron–glia communication after the initiating infection has resolved or diminished. PRRs expressed in glial cells allow microbial signals to trigger inflammatory and, subsequently, danger-related signals to drive a pathological neuroinflammatory state involved in PIPP [8]. Through these responses, glial cells amplify neuronal excitability, disturb synaptic homeostasis, and promote central sensitization, thereby contributing to the transition from transient inflammatory or infection-associated signals to persistent pain states [10, 22, 23]. Accordingly, glial activation provides a plausible cellular basis for the transition from infection-triggered inflammation to self-sustaining neuroinflammation, explaining how pain may persist even after overt pathogen burden has declined.

### Epithelial cells shape the peripheral neuroimmune environment

Epithelial cells are positioned at host barrier interfaces where persistent pathogen, retained microbial ligands, or DAMPs can prolong peripheral neuroimmune signaling. Rather than serving solely as structural barriers, epithelial cells express PRRs and respond to microbial signals by regulating the release of inflammatory mediators, which recruit immune cells and shape local inflammatory responses, and they can also respond to DAMPs that prolong sterile inflammation rather than serving only as structural barriers [9]. For example, in intestinal epithelial cells, the commensal-derived membrane protein Amuc_1100 from *Akkermansia muciniphila* interacts with TLR2 and enhances mucus thickness and tight junction protein expression, illustrating that epithelial PRR signaling can regulate barrier integrity as well as host defense functions [31]. By shaping local inflammation and tissue integrity, epithelial cells can indirectly influence nearby sensory pathways and help maintain peripheral sensitization that favors PIPP. Thus, epithelial cells should be viewed as active participants in prolonged peripheral neuroimmune communication, especially at sites where host tissues remain exposed to microbial products.

## How microbial sensing becomes persistent neuroimmune signaling

Although microbial PAMPs initiate innate immune responses during most infections, their capacity to drive PIPP varies considerably among pathogens. This variability depends not only on the molecular nature of individual PAMPs, but also on whether microbial signals persist after acute infection through retained microbial products, viral latency, recurrent antigen exposure, or long-lasting immune reprogramming. Consequently, different pathogen classes engage distinct upstream mechanisms that converge on persistent PRR-mediated neuroimmune activation.

### Persistent bacterial PAMPs sustain neuroimmune signaling

Recent studies have extended classical bacterial PRR biology by showing that retained bacterial PAMPs may continue to drive neuroimmune signaling after the acute phase of infection. Bacterial pathogens contain a diverse repertoire of PAMPs that activate Toll-like receptors (TLRs), NOD (nucleotide oligomerization domain)-like receptors, and inflammasomes, leading to sustained neuroimmune signaling that promotes nociceptor sensitization and PIPP. Lipopolysaccharide (LPS), a major component of the outer membrane of Gram-negative bacteria, is recognized by TLR4 in association with MD-2 (myeloid differentiation factor 2) and CD14 (cluster of differentiation 14) [32]. In primary sensory neurons, LPS–TLR4 signaling may engage downstream pro-inflammatory pathways and could contribute to enhanced TRPV1 (transient receptor potential vanilloid 1)-mediated nociceptor excitability [33, 34]. In addition, TLR4 endocytosis triggers TRIF (TIR-domain-containing adapter-inducing interferon-β)-dependent signaling that induces type I interferon (IFN) responses that further sensitize nociceptors and activate spinal microglia [33]. In multiple infection models, LPS-associated hypersensitivity can persist for weeks and has been associated with delayed clearance or sequestration of LPS in tissues [35].

Gram-positive bacteria employ distinct PAMPs. Lipoteichoic acid (LTA) is recognized by TLR2 and may engage TLR2/TLR6-related signaling, recruiting canonical MyD88-dependent pathways that contribute to pro-nociceptive neuroinflammatory responses [64, 65]. Peptidoglycan fragments are sensed by NOD1 and NOD2, which signal through receptor-interacting serine/threonine protein (RIP2) and downstream p38-related pathways. Given that p38 signaling has been linked to increased excitability and sodium channel function in sensory neurons, this pathway may also contribute to mechanical allodynia [66, 67].

Other bacterial components such as CpG (Cytosine-phosphate-Guanine) DNA can activate TLR9-dependent signaling which may influence inflammatory responses relevant to neuroimmune sensitization [62]. In addition, flagellin, recognized by TLR5, has been implicated in promoting A-fiber hypersensitivity and mechanical allodynia, further supporting a role for bacterial PAMPs in PRR-mediated pain amplification [36]. Collectively, bacterial infections illustrate how retained microbial ligands can persist as active inflammatory cues, rather than passive markers of prior infection, thereby sustaining pain-promoting neuroimmune signaling after pathogen clearance.

### Viral latency and persistent viral nucleic acid sensing sustain neuroimmune activation

Viral infections generate nucleic acid–based PAMPs that are sensed by endosomal and cytosolic PRRs, driving IFN signaling and neuroimmune interactions that may contribute to PIPP. Double-stranded RNA activates endosomal TLR3 as well as the cytosolic sensors retinoic acid-inducible gene I (RIG-I) and melanoma differentiation-associated protein 5 (MDA5), whereas single-stranded RNA is recognized by TLR7 and TLR8, and cytosolic viral DNA is detected by cGAS (cyclic GMP-AMP synthase) [20, 68].

Compared with many bacterial ligands, viral PAMPs are particularly relevant to persistent pain because latency, tissue reservoirs, and incomplete clearance can prolong nucleic acid sensing over extended periods. In recent decades, post-viral syndromes have revived interest in how persistent viral PAMPs sustain neuroimmune activation. Viral latency represents a key mechanism by which viral nucleic acids persist within host tissues and continue to engage innate immune sensing pathways. Varicella-zoster virus (VZV) establishes lifelong latency in sensory ganglia characterized by highly restricted viral transcription [37]. During latency, this restricted viral transcription may generate nucleic acid species capable of engaging cytosolic RNA sensors such as RIG-I and MDA5, which can predispose nociceptor sensitization and the development of PHN [38, 39]. Upon reactivation, abundant viral PAMPs may promote neuronal injury, potentially contributing to the severity and persistence of PHN [40, 41]. Persistent SARS-CoV-2 reservoirs have also been proposed [44]. The SARS-CoV-2 spike protein can engage TLR4 to trigger neuroimmune signaling, and persistent SARS-CoV-2 reservoirs in the gut or in host cells have been proposed to serve as a continued source of viral RNA or protein PAMPs which may sustain PRR activation and could contribute to persistent pain in a subset of patients with long COVID [44, 45]. Together, these observations suggest that virus-driven PIPP may arise even in the absence of productive viral replication. Persistent viral sensing through latent transcription or residual viral products can remain biologically active, suggesting how microbial sensing is linked to long-term neuroimmune activation and persistent pain.

### Repeated fungal and parasitic PAMP exposure promotes durable neuroimmune imprinting

Compared with bacterial and viral infections, direct mechanistic evidence linking fungal or parasitic PAMPs to PIPP remains comparatively limited. Nevertheless, these pathogens illustrate how persistent or recurrent innate immune stimulation, barrier disruption, fibrosis, and prolonged neuroimmune remodeling may create conditions permissive for PIPP. Fungal and parasitic infections are often characterized by incomplete pathogen clearance, resulting in persistent or recurrent antigen exposure that intermittently reactivates PRR-dependent neuroimmune signaling and sustains pain [47]. The pathogenic relevance of PAMPs may lie less in rapid acute sensing and more in their capacity to repeatedly reinforce inflammatory programs that favor PIPP.

Fungal pathogens expose cell wall–derived PAMPs that are primarily sensed by C-type lectin receptors (CLRs) and selected TLRs, shaping antifungal immune responses and Th17-associated programs [48, 49]. β-glucan activates Dectin-1 on myeloid cells, inducing spleen tyrosine kinase-caspase recruitment domain-containing protein 9-nuclear factor kappa B (Syk-CARD9-NF-κB) signaling and IL-23 production, while also promoting trained immunity that enhances inflammatory responsiveness [48, 49]. IL-23 supports Th17 differentiation, and T helper cell (Th) 17-derived IL-17A can act directly on sensory neurons to enhance nociceptor excitability and mechanical hypersensitivity [18]. Chitin can engage innate immune receptors, most clearly TLR2 and in some contexts Dectin-1, to induce inflammatory cytokines and other mediators; however, its effects are context-dependent and likely act in coordination with other fungal PAMPs rather than as a dominant independent driver [50, 51].

Helminths elicit complex immune responses. For example, schistosome egg-derived ligands are classically associated with dominant Th2 polarization while also engaging innate immune pathways, including TLR4-associated signaling and NOD-like receptor family pyrin domain-containing protein 3 (NLRP3) inflammasome activation; NLRP3-dependent IL-1β production contributes to fibrosis and may help create a pro-nociceptive inflammatory milieu associated with visceral hypersensitivity [52–54, 69]. Thus, across fungal and parasitic infections, persistent or repetitive exposure to pathogen-derived ligands and tissue-derived DAMPs may help stabilize PIPP-promoting immune states even when direct pathogen or debris burden is low or fluctuating.

Although bacterial, viral, fungal, and parasitic pathogens differ in biology, they converge on shared neuroimmune mechanisms relevant to PIPP. Bacterial infections highlight persistent inflammatory ligands, viral infections prolonged microbial sensing, fungal infections trained innate immunity, and parasitic diseases chronic immune remodeling. These mechanisms are complementary rather than exclusive, and their contributions likely vary by pathogen, tissue, and disease stage. This pathogen-centered view provides the context for how diverse upstream microbial stimuli converge on common downstream PRR-mediated neuroimmune programs discussed below.

## Convergent neuroimmune programs downstream of PAMP-PRR signaling

Although PRRs recognize diverse microbial ligands, their downstream signaling pathways often engage conserved neuroimmune programs that shape persistent pain. PAMPs are detected by PRRs expressed predominantly by innate immune cells and glial cells, and in certain contexts by sensory neurons themselves [70, 71]. Distinct PRR families can drive overlapping inflammatory, neuronal, and glial responses that enhance nociceptor sensitization and sustain neuron–immune–glial communication beyond acute infection. The outcome of PRR activation is further shaped by cellular context, as the same microbial ligand may elicit distinct responses depending on whether PRRs are engaged in immune cells, sensory neurons, glial cells, or epithelial tissues [70, 72]. Thus, the significance of PAMP–PRR signaling in PIPP lies not only in pathogen recognition, but also in its ability to translate diverse microbial inputs into pain-relevant neuroimmune mechanisms.

### PRRs as neuroimmune sensing systems

PRRs comprise several major receptor families that differ in cellular localization and ligand specificity, including TLRs, nucleotide-binding oligomerization domain-like receptors (NLRs), retinoic acid-inducible gene I-like receptors (RLRs), CLRs, and absent in melanoma 2 (AIM2)-like receptors (ALRs) [68, 70]. These receptor families form a core innate sensing network that detects infection-associated microbial products and initiates host-defense responses.

Although these receptor classes detect distinct microbial ligands, they are united by their ability to trigger inflammatory signaling, shape neuroimmune communication, and promote nociceptor sensitization and persistent pain. In PIPP, PRRs therefore function not only as microbial sensors but also as part of an integrated neuroimmune sensing system through which diverse pathogens engage shared pain-relevant downstream responses.

### Shared downstream programs of PRR activation

Recent studies suggest that PRR signaling across immune and non-immune cellular compartments contributes not only to pathogen clearance but also to inflammatory and neuroimmune programs that can promote nociceptor sensitization and persistent pain. Although distinct PRRs recognize different microbial ligands, their downstream pathways often converge on shared signaling outputs that are highly relevant to PIPP. This convergence provides a mechanistic basis for why bacterial, viral, fungal, and parasitic infections, despite major differences in pathogen biology, may produce overlapping persistent pain phenotypes [7, 14].

#### Convergent inflammatory signaling

A major downstream consequence of PRR activation is the induction of inflammatory transcriptional programs that connect microbial sensing to pain-relevant neuroimmune responses. Recognition of PAMPs by PRRs in immune cells triggers the production of cytokines, chemokines, and lipid mediators. TLRs signal mainly through the adaptor proteins MyD88 or TRIF, with MyD88-dependent pathways activating NF-κB and MAPK (mitogen-activated protein kinase) signaling to drive transcription of pro-inflammatory mediators [68, 73]. NOD1 and NOD2 signal through RIP2 kinase, whereas CLR signaling proceeds through Syk and CARD9 to NF-κB, further reinforcing inflammatory gene expression and mediator release [73].

In parallel, antiviral and IFN-linked pathways represent another important component of this convergent response. TRIF-dependent signaling leads to the activation of TBK1 (TANK-binding kinase 1) and interferon regulatory factor 3 (IRF3), inducing type I IFN responses [42]. Similarly, the cytosolic viral RNA sensors RIG-I and MDA5 signal through MAVS (mitochondrial antiviral-signaling protein) to activate IRF3 and related antiviral transcriptional programs [43]. Although these pathways are classically discussed in the context of host defense, they may also influence glial reactivity and neuronal function in ways that contribute to persistent pain.

Inflammasome activation provides an additional route by which upstream PRR signaling is translated into potent effector cytokine production. NLRP3 and AIM2 (absent in melanoma 2) assemble inflammasomes via ASC (Apoptosis-associated speck-like protein containing a caspase recruitment domain) to activate caspase-1 and promote IL-1β and IL-18 maturation [56]. Mature inflammatory cytokines such as IL-1β are well-established drivers of nociceptor sensitization and neuroinflammation. Together, these pathways show that diverse PRRs engage a limited repertoire of inflammatory programs that can sustain pain-relevant neuroimmune signaling.

####  Celluar execution of neuroimmune amplification

These molecular programs are implemented across multiple cellular compartments, where they are translated into neuroimmune signals that promote nociceptor sensitization and persistent pain. Sensory neurons can directly detect microbial ligands through functional PRRs expressed in dorsal root ganglia and trigeminal ganglia. In nociceptors, TLR4 recognizes LPS, TLR2 detects bacterial lipopeptides and LTA, and TLR3 senses viral double-stranded RNA [11, 21, 57]. Cytosolic NOD1 and NOD2 sense peptidoglycan-derived fragments and signal through RIP2 [66]; Notably, p38 signaling in sensory neurons has been associated with increased excitability and modulation of the sodium channel Nav1.8 [67]. Beyond directly increasing excitability, neuronal PRR activation can also promote the release of neuropeptides such as SP and CGRP. This may promote bidirectional neuroimmune crosstalk through interactions with local immune cells, potentially amplifying neuroinflammation and contributing to peripheral sensitization [58, 63].

Glial cells further amplify PIPP-relevant outputs and help sustain neuroinflammatory circuits. Satellite glial cells surrounding sensory neuron somata contribute to ganglionic neuroimmune signaling and can promote neuronal sensitization through the release of inflammatory mediators [59]. Schwann cells similarly express TLRs and other PRRs and, upon activation, produce inflammatory mediators that influence axonal function and pain signaling [74]. In the spinal cord, microglia release factors such as IL-1β and TNF that enhance excitatory synaptic transmission and may further drive central sensitization [7], while astrocytes sustain neuroinflammatory signaling through cytokines and gliotransmitters [24, 25]. Together, these glial responses help convert transient infection-related signals into more durable neuron–glia inflammatory loops.

Epithelial cells also contribute to this amplification by coupling innate sensing to local neuroactive mediator release. Epidermal keratinocytes and intestinal epithelial cells express multiple PRRs, including TLR3 and TLR4 [9, 13, 63]. Upon activation by microbial PAMPs or damage-associated signals, these cells release mediators such as thymic stromal lymphopoietin, IL-33, and endothelin-1 (ET-1), which can directly act on nearby sensory endings [75]. Thus, epithelial tissues not only detect infection but also actively shape a peripheral signaling environment that favors persistent nociceptor hyperexcitability.

Taken together, these observations indicate that PRR activation across immune, neuronal, glial, and epithelial compartments converges onto a set of shared PIPP-relevant outcomes, including inflammatory mediator release, nociceptor sensitization, glial activation, and amplification of peripheral and central sensitization.

### Mechanisms sustaining PRR signaling beyond infection

A key implication of the PAMP–PRR model is that infection-induced innate immune signaling may persist beyond the apparent resolution of acute infection. This may reflect retained microbial ligands, continued sensing of latent or restricted viral products, or durable innate immune reprogramming such as trained immunity [35, 37–41, 44–46, 48, 49, 55]. These mechanisms are not mutually exclusive and may represent sequential phases of PIPP. Early pain persistence may still be supported by persistent pathogen or microbial debris, whereas later stages may be maintained by hyper-responsive innate immunity, DAMP-driven sterile neuroinflammation, and self-reinforcing neuron–glia–immune circuits. PRRs should therefore be considered not only as pathogen sensors, but also as molecular gateways through which transient infection is translated into persistent pain-promoting neuroimmune states.

## Therapeutic implications: targeting persistent neuroimmune programs rather than infection alone

At present, no therapy has been established specifically for PIPP by directly targeting PAMP–PRR signaling. Nevertheless, recent advances in neuroimmunology provide a mechanistic rationale for considering new interventions. While preclinical evidence supporting innate immune targeting is increasing, most data come from general inflammatory or neuropathic pain models rather than PIPP-specific settings, and clinical translation remains uncertain.

### Mechanism-guided targeting of PRR-mediated neuroimmune signaling

Recent preclinical studies have shifted from broad immunosuppression to more selective pharmacological targeting of PRR downstream effectors such as NLRP3 inflammasome. Pharmacological and genetic studies demonstrate that specific NLRP3 inhibitors and genetic targeted approaches effectively block NLRP3 inflammasome activation and its downstream signaling components, such as microglial IL-1β release [26, 27]. Although derived mainly from inflammatory and neuropathic pain models, these findings suggest that NLRP3-driven neuroimmune amplification may also contribute to PIPP, particularly when residual microbial products or sterile inflammation persist. However, the therapeutic potential of targeting NLRP3 in PIPP remains plausible but has not yet been established [12].

Similarly, targeting TLR4 at the neuroglial interface continues to show preclinical efficacy in the treatment of neuropathic pain. Small-molecule modulators such as ACT001 and lovastatin have been reported to target the TLR4 co-receptor MD-2, thereby suppressing downstream TLR4/MyD88 signaling, reducing glial activation and neuroinflammatory responses, and improving pain behaviors in preclinical neuropathic pain models [28, 29]. These observations support the broader concept that PRR-directed modulation may attenuate maladaptive neuroimmune signaling, although direct evidence in PIPP remains limited. In addition, interest has grown in specific microbial sensing pathways that may also influence nociception. The stimulator of interferon genes (STING) pathway, canonically known for detecting pathogenic DNA during infections, has emerged as a key modulator. Recent evidence demonstrates that activating, rather than blocking, STING in sensory neurons elevates IFN-β levels, which subsequently decreases nociceptor action potential firing [20]. However, the effects of STING modulation are likely cell-type- and context-dependent, and current evidence is largely limited to preclinical rather than PIPP-specific models. Accordingly, although STING modulation is mechanistically intriguing and may have particular relevance in infection-associated contexts, its utility as a therapeutic strategy for PIPP remains speculative at present [30]. Overall, these findings support the plausibility of targeting PRR-mediated neuroimmune networks in persistent pain, but in PIPP such approaches remain hypothesis-generating rather than validated therapies.

### From pathway inhibition to resolution-oriented precision therapy

Despite preclinical successes, PRR antagonists may fail in persistent pain clinical trials due to off-target effects, redundant inflammatory pathways, and severe immunosuppressive risks. Translating the mechanistic insights into clinical practice requires more cautious and refined strategies.

First, promoting active neuroimmune resolution. Rather than relying solely on prolonged pathway inhibition, utilizing specialized pro-resolving mediators (SPMs) may represent a complementary or alternative approach. SPMs have been shown in preclinical studies to promote a phenotypic switch in hyper-responsive microglia and macrophages at the neuroglial interface, enhancing their phagocytic capacity to clear residual microbial debris [19, 58]. These actions may attenuate downstream neuroinflammation while preserving broader host defense. However, evidence supporting this strategy in PIPP remains indirect, and current support comes mainly from experimental models of neuroinflammation or pain rather than from PIPP-specific studies [19, 58].

Second, biomarker-driven patient stratification. It is essential to validate fluid and exosomal biomarkers, including circulating NLRP3 inflammasome components and neurofilament light chains [76–78]. Such biomarkers may help distinguish active PAMP-driven neuroinflammation from structural nerve damage and improve trials of targeted interventions in biologically enriched groups.

Third, matching therapy to mechanism and timing. If residual microbial ligands or viral reservoirs continue to drive PRR activation, reducing ligand sensing may be appropriate. By contrast, when trained immunity, glial activation, and DAMP-driven sterile neuroinflammation predominate, resolution-oriented or circuit-disrupting approaches may be more rational than prolonged upstream PRR blockade. Although this working model remains provisional, it may help guide future mechanistic studies and clinical trial design in PIPP.

## Conclusion

Persistent pain may continue or recur after clinical resolution of the infection and should not, by itself, be interpreted as evidence of ongoing infection. Although residual tissue damage, fibrosis, or irreversible nerve injury may contribute to ongoing pain, these factors do not fully explain pain persistence after pathogen clearance. Instead, microbial PAMP–PRR signaling may provide a mechanistic link between transient infection and sustained neuroimmune activation.

Although distinct pathogens activate different PAMP–PRR pathways, their ligands may converge on shared neuroimmune programs involving immune cells, sensory neurons, glia, and epithelial cells. These programs can drive inflammatory amplification, neuron–immune crosstalk, glial activation, and sensitization of pain pathways. Accordingly, persistent pain after infection should not be equated with active infection, because PRR signaling may be sustained by retained microbial products, products derived from latent pathogens, recurrent antigen exposure, trained innate immunity, or a transition to DAMP-mediated sterile neuroinflammation, depending on the pathogen, tissue, and disease context.

Therapeutic translation should emphasize mechanism- and timing-guided strategies that promote neuroimmune resolution, rather than relying solely on pathogen eradication or prolonged PRR blockade. Overall, this review highlights how microbial PAMP–PRR sensing can evolve from an acute host-defense response into persistent neuroimmune signaling involving immune, neuronal, glial, and epithelial cells, thereby driving nociceptor sensitization and PIPP.

## Data Availability

Not applicable.

## Abbreviations

CARD9: Caspase recruitment domain-containing protein 9
CGRP: Calcitonin gene-related peptide
DAMPs: Damage-associated molecular patterns
IFN: Interferon
IL: Interleukin
IRF3: Interferon regulatory factor 3
LTA: Lipoteichoic acid
LPS: Lipopolysaccharide
MDA5: Melanoma differentiation-associated protein 5
NF-κB: Nuclear factor kappa B
NLRP3: NOD-like receptor family pyrin domain-containing protein 3
PAMP: Pathogen-associated molecular pattern
PHN: Postherpetic neuralgia
PIPP: Post-infectious persistent pain
PRR: Pattern recognition receptor
RIG-I: Retinoic acid-inducible gene I
RIP2: Receptor-interacting serine/threonine protein
SP: Substance P
STING: Stimulator of interferon genes
Syk: Spleen tyrosine kinase
Th T: Helper cell Th
TLR: Toll-like receptor
TNF: Tumor necrosis factor
TRPA1: Transient receptor potential ankyrin 1
